# Benefits of treadmill training for patients with Down Syndrome

**DOI:** 10.1192/j.eurpsy.2022.970

**Published:** 2022-09-01

**Authors:** K. Kamińska, M. Ciołek, K. Krysta

**Affiliations:** 1 Medical University of Silesia, Students` Scientific Association, Department Of Rehabilitation Psychiatry, Katowice, Poland; 2 Medical University of Silesia, Department Of Rehabilitation Psychiatry, Katowice, Poland

**Keywords:** treadmill training, intellectual disability, Down syndrome

## Abstract

**Introduction:**

Down syndrome (DS) is a complex condition that causes various health problems and it is accepted that treadmill training is a therapy method for some of them.

**Objectives:**

The objective was to evaluate the effectiveness of various results of treadmill training in children and adults with DS.

**Methods:**

We included studies in which participants with DS from every age group received treadmill training, alone or combined with physiotherapy and could optionally be compared to a control group with patients with DS who did not use treadmill training. The search was conducted in medical databases: PubMed, PEDro, Science Direct, Scopus and Web of Science and involved trials published until July 2021. Following PRISMA criteria, the Risk of Bias assessment was conducted using a tool developed by the Cochrane Collaboration for RCT. The included studies presented multiple outcomes and various methodologies therefore we were not able to conduct any sort of data synthesis, we presented measures of treatment effect as mean differences and corresponding 95% confidence intervals.

**Results:**

5 studies with a total number of 687 participants were included. 10 trials reported on walking onset, 8 on gait parameters or cardiovascular functions, 4 on anti-inflammatory effect and 3 on executive or cognitive functions. We came across 25 different outcomes in different age groups which are presented in a narrative manner. In all outcomes we have observed a positive result favouring the treadmill training.

**Conclusions:**

Introducing treadmill exercise into typical physiotherapy generates improvements of mental and physical health of people with DS of all ages.

**Disclosure:**

No significant relationships.

